# Biophysical rationale and emerging clinical perspectives of surfactants in thoracic surgery: a translational review

**DOI:** 10.1007/s00423-026-04265-2

**Published:** 2026-09-29

**Authors:** Xiayimaierdan Yibulayin, Ehsan Arkin, Guangmin Yang, Sebastian Kalverkamp, Jan W. Spillner, Johannes Greven

**Affiliations:** Department of Thoracic Surgery, University Clinic RWTH Aachen, Pauwelsstraße 30, Aachen, 52074 Germany

**Keywords:** Surfactant, Thoracic surgery, Lung compliance, ARDS, Drug delivery, Biosurfactant, Intraoperative care

## Abstract

**Background:**

Pulmonary surfactants are essential for alveolar stability and gas exchange. While their clinical use is well established in neonatal care, their potential in thoracic surgery remains underexplored.

**Objective:**

To review the current and emerging applications of surfactants in thoracic surgical practice, and to assess their role in perioperative lung protection.

**Methods:**

We reviewed recent literature on surfactant biology, perioperative administration strategies, and novel formulations, including bio-derived and responsive surfactants.

**Results:**

Exogenous surfactants may improve lung compliance, preventing atelectasis, and mitigating postoperative respiratory complications. In thoracic surgery, surfactants may also serve as drug delivery vehicles or anti-inflammatory agents. However, clinical use remains limited, and further studies are needed to define optimal dosing, timing, and patient selection.

**Conclusions:**

Surfactants represent a versatile and evolving tool in thoracic surgery. With continued innovation and clinical validation, they may become an effective adjunct for enhancing perioperative respiratory outcomes.

## Introduction

Surfactants, or surface-active agents, are amphiphilic molecules capable of reducing surface tension at the interface between different phases [1]. In biologic systems, pulmonary surfactants are essential for maintaining stability of alveolar by lowering the surface tension of the fluid lining the alveoli [2]. This prevents alveoli collapse during expiration and ensures efficient gas exchange [3]. Their ability to modify interfacial properties makes them indispensable in respiratory physiology and targeted biomedical applications.

While the role of surfactants in neonatal respiratory distress syndrome (NRDS) has been well established [4–5], its efficacy in adult populations remains unclear. Despite the clear biophysical rationale, recent meta-analyses of randomized controlled trials have indicated that exogenous surfactant administration does not significantly improve clinical outcomes, such as mortality, in adult patients with acute respiratory distress syndrome (ARDS) [6]. However, unlike the unpredictable onset and widespread systemic destruction of medical ARDS, surgical lung injury follows a predictable timeline, offering a crucial window for early intervention. Procedures routinely involve controlled, temporary lung deflation, primarily through one-lung ventilation (OLV). The pathophysiology of OLV and the subsequent development of postoperative pulmonary complications represent a multifaceted process that extends far beyond simple mechanical alveolar collapse. While impaired compliance and atelectasis are significant physiological precursors, they are part of a broader cascade exacerbated by profound variations in pulmonary perfusion. Specifically, the interplay between hypoxic pulmonary vasoconstriction (HPV) and ventilation-perfusion (V/Q) mismatch during OLV creates a localized environment of hypoxia and microvascular stress [7]. Upon lung re-expansion, ischemia-reperfusion injury triggers the release of reactive oxygen species and inflammatory mediators, which directly impair the metabolic machinery of Type II alveolar epithelial cells [8]. Furthermore, increased capillary permeability leads to the leakage of plasma proteins—such as albumin and fibrinogen—into the alveolar space, where they competitively inactivate existing surfactants [9]. Consequently, the clinical reality of Postoperative Pulmonary Complications (PPCs) is driven by this synergistic effect of mechanical, vascular, and inflammatory insults. Therefore, the potential for surfactant-based interventions in thoracic surgery should be viewed through the lens of targeted proactive perioperative lung protection rather than reactive treatment for established respiratory failure. Therefore, the potential for surfactant-based interventions in thoracic surgery needs further study. This review aims to provide a comprehensive overview of surfactants in the context of thoracic surgery, bridging the gap between their biophysical properties and their clinical perspectives. We also highlight emerging innovations and challenges.

## Biophysical properties of surfactants

Pulmonary surfactants are a lipid-rich complex essential for alveolar stability and efficient gas exchange. It typically consists of approximately 90% of lipids and 10% of surfactant-associated proteins, with phosphatidylcholine (PC) as the dominant lipid, accounting for 80% of total phospholipids—half of which are disaturated species such as dipalmitoylphosphatidylcholine (DPPC) [10–13]. Acidic phospholipids including phosphatidylglycerol (PG), phosphatidylinositol (PI), and bis(monoacylglycero)phosphate constitute 15% of the lipid pool, while minor components such as phosphatidylethanolamine (PE), sphingomyelin (SM), and lysophosphatidylcholine (LPC) contribute to surfactant diversity [11–12]. Surfactant proteins (SP-A, SP-B, SP-C, SP-D) synergistically interact with lipids to reduce surface tension and facilitate rapid re-spreading during respiration [11, 14–15]. These lipids and proteins act synergistically at the air–liquid interface of alveoli to reduce surface tension to near-zero levels during exhalation, preventing alveolar collapse, and to facilitate rapid re-spreading upon inhalation [11]. Surfactant molecules are amphiphilic, comprising a hydrophilic head and a hydrophobic tail, which enables their orientation and accumulation at the interface between aqueous and gaseous phases in the lung [16–17]. This interfacial behavior is fundamental to maintaining dynamic alveolar function throughout the respiratory cycle.

**Fig. 1:**
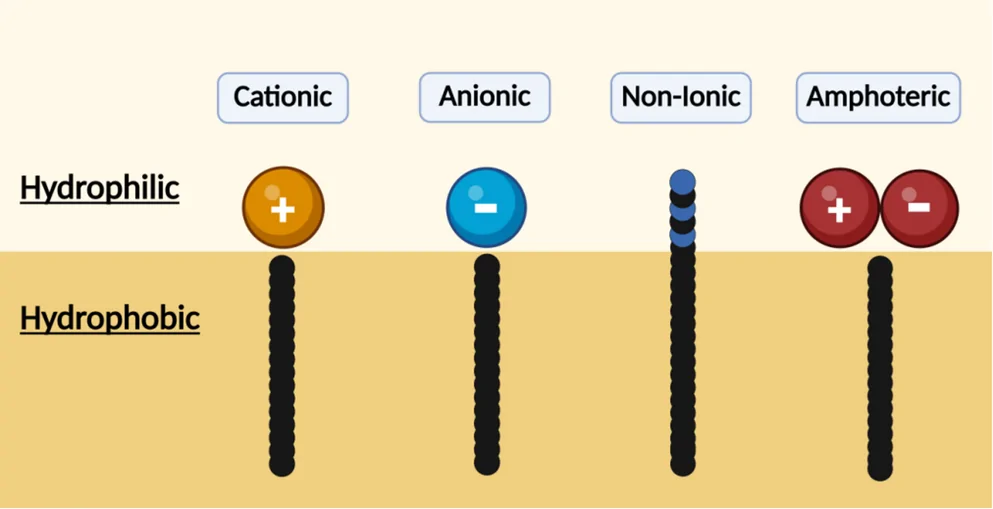
Structural classification of surfactants based on hydrophilic head group polarity. Surfactants are amphiphilic molecules composed of a hydrophilic (water-attracting) head and one or more hydrophobic (lipid-soluble) tails. Depending on the charge of the head group, surfactants are categorized as: Cationic, carrying a positive charge (e.g., quaternary ammonium compounds); Anionic, carrying a negative charge (e.g., sodium dodecyl sulfate); Non-ionic, lacking charge but containing polar functional groups (e.g., poloxamers); Amphoteric, possessing both positive and negative charges depending on pH (e.g., betaine)

As shown in Fig. 1, surfactants are broadly classified based on the nature of their hydrophilic head group. This determines their electrostatic interactions, cytotoxicity, and potential for pulmonary application. In particular, non-ionic and amphoteric surfactants are considered more biocompatible for respiratory formulations due to their low irritation potential and flexible interfacial behavior. In the broader context of biomedical surfactants—including those used in synthetic or drug delivery systems—classification is based on the net charge of the hydrophilic head group. Surfactants fall into four major categories [18–22]: (1) Anionic surfactants, which possess strong interfacial activity but are often cytotoxic and unsuitable for pulmonary use. (2) Cationic surfactants, valued for their antimicrobial properties but limited by high toxicity. (3) Nonionic surfactants, which are widely used for their biocompatibility and low irritancy. (4) Zwitterionic (amphoteric) surfactants, which contain both positive and negative charges and exhibit pH-dependent behavior, offering a balanced profile of activity and tolerability.

Recent experimental advances have significantly enhanced our molecular understanding of pulmonary surfactant function. Tools such as the constrained drop surfactometer (CDS) [23–24], fluorescence microscopy [25], X-ray diffraction [26], and molecular dynamics simulations [27] have revealed key features of lipid monolayer formation, interfacial fluidity, and surfactant film compressibility. Upon adsorption, a monolayer enriched in DPPC and cholesterol forms rapidly at the air-liquid interface [28]. This stable structure resists collapse during exhalation through an adsorption-driven lipid sorting process. During deep inhalations or sighs, transient monolayer disruption may occur, triggering a compression-driven sorting mechanism that restores the functional surfactant film [29]. Surface tension in the alveolar lining arises from cohesive forces among water molecules. Pulmonary surfactant reduces this tension by disrupting hydrogen bonding, thereby lowering the energy required for alveolar inflation—particularly important during expiration [30]. These principles are directly relevant to thoracic surgery, where alveolar de-recruitment and surfactant inactivation often lead to impaired postoperative lung function [31].

An in-depth understanding of surfactant biophysics provides a foundation for the development of synthetic or recombinant formulations aimed at restoring or enhancing intraoperative pulmonary mechanics. Tailoring surfactant composition to closely mimic physiological conditions may optimize alveolar recruitment, reduce ventilator-induced lung injury, and improve postoperative outcomes in thoracic surgical patients.

## Pulmonary surfactant physiology and pathophysiology

### Homeostasis and the role of alveolar type II cells

While the biophysical function of pulmonary surfactant is well understood, its dysfunction plays a critical role in the pathogenesis of several pulmonary diseases and presents unique challenges in thoracic surgical settings. Surfactant abnormalities may result from insufficient synthesis, altered lipid–protein composition, or inactivation by plasma proteins and inflammatory mediators [32]. The restoration and maintenance of surfactant homeostasis depend heavily on alveolar type II (AE-II) epithelial cells. These specialized cells synthesize surfactant lipids and proteins within lamellar bodies, secrete them into the alveolar space, and mediate their re-uptake and recycling through regulated endocytosis [33–34]. This recycling process is critically modulated by surfactant proteins—particularly SP-A and SP-D—which facilitate lipid uptake and protect against inflammation [35].

### Pathological mechanisms

**Fig. 2:**
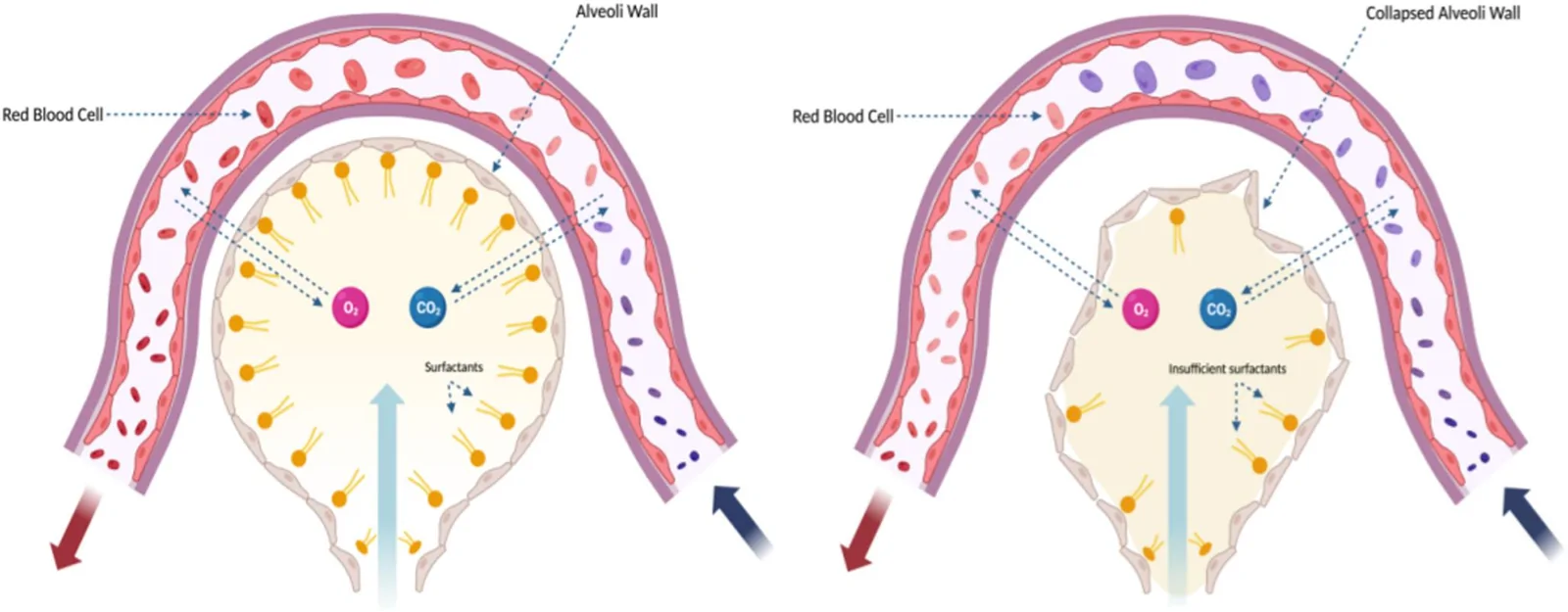
Comparison between normal and NRDS alveoli: effects of surfactant sufficiency versus deficiency. The left panel illustrates a normal alveolus lined with adequate pulmonary surfactants. These amphiphilic molecules form a monolayer at the air-liquid interface, reducing surface tension, stabilizing alveolar architecture, and facilitating efficient gas exchange across a thin alveolar-capillary membrane. In contrast, the right panel represents an alveolus affected by NRDS, characterized by surfactant deficiency. The collapsed alveolar walls, reduced compliance, and impaired gas exchange are evident. Inadequate surfactant levels result in increased surface tension, alveolar instability, and reduced oxygen uptake, contributing to respiratory failure in preterm infants

As illustrated in Fig. 2, severe surfactant deficiency leads to increased alveolar surface tension and structural collapse, hallmark features of NRDS. Compared to the surfactant-rich, compliant alveoli seen in normal physiology, NRDS lungs exhibit impaired gas exchange and alveolar derecruitment due to loss of interfacial stabilization. In NRDS, immature alveolar type II cells fail to produce adequate surfactant, leading to widespread atelectasis [36]. Surfactant replacement therapy has revolutionized neonatal care and remains a mainstay treatment [37–38]. In adults, ARDS is similarly marked by surfactant inactivation due to inflammatory exudation and oxidative stress [39]. However, in the distinct context of thoracic surgery, surfactant impairment is largely mechanically driven and surgically induced [40–43]. Primarily, OLV—the standard for nearly all non-cardiac thoracic procedures—represents the most significant and universal damage to the surfactant system. During the collapse phase of OLV, surfactants are displaced from the air-liquid interface, leading to their conversion from functional large aggregates (LA) into inactive small aggregates (SA) [7]. Upon lung re-expansion, the high surface tension in these unstable alveoli necessitates significant opening pressures, triggering mechanical shear stress that further damages the alveolar-capillary membrane [44]. This “atelectrauma” is often accompanied by the leakage of plasma proteins, such as albumin and fibrinogen, which competitively inhibit surfactant adsorption and activity. By prioritizing the study of OLV-induced surfactant changes, researchers can target the most prevalent driver of PPCs.

### The paradigm shift: from “bystander” to “culprit”

A critical consideration is whether surfactant dysfunction acts as a primary culprit or a secondary bystander in the pathogenesis of surgical lung injury. While surfactant depletion often initiates as a “bystander” consequence of inflammatory exudation and plasma protein leakage, the resulting loss of interfacial stability rapidly evolves into a “culprit” by triggering a vicious cycle [45]. The repetitive collapse and re-expansion of unstable alveoli induce mechanical biotrauma, which exacerbates the underlying inflammatory response and further impairs surfactant turnover. Unlike adult ARDS trials where treatment is typically reactive, thoracic surgery provides a unique prophylactic window for intervention. Administering surfactants prior to or during the initial stages of lung re-expansion after OLV may prevent the onset of this biomechanical cascade, rather than merely serving as a symptomatic remedy for established respiratory failure [46]. Furthermore, surfactants should be viewed as a multifunctional therapeutic platform. Beyond their biophysical role, surfactant-associated proteins exert crucial immunomodulatory effects, while the lipid matrix can function as a specialized carrier for targeted drug delivery [47]. This integrated approach shifts the rationale from simple replacement therapy toward a comprehensive, perioperative lung-protective strategy.

## Exogenous surfactant therapy: clinical experience

### The evolution of surfactant formulations

Exogenous surfactant therapy is well-established treatment for NRDS, where endogenous production is deficient due to lung immaturity. Since the 1990s, clinical administration of surfactants has significantly reduced mortality and respiratory complications in preterm infants [10]. Several formulations have been approved for neonatal use, including both animal-derived and synthetic surfactants. Animal-derived surfactants, such as beractant (bovine origin), poractant alfa (porcine origin), and calfactant, are extracted from minced lung tissue or alveolar lavage fluids [48–49]. They contain both phospholipids and surfactant proteins, which contribute to their high efficacy in restoring alveolar stability [11]. On the other hand, synthetic surfactants, such as lucinactant, are composed primarily of DPPC and synthetic peptides that mimic the function of SP-B. While synthetic formulations offer advantages in terms of batch consistency, reduced risk of immunogenicity, and greater production scalability, they often lack the full biochemical complexity of natural surfactant systems, which may impact their overall performance in vivo [50–51].

### The adult ARDS bottleneck and the thoracic pivot

In contrast to neonates, where surfactant deficiency is a primary issue, adult respiratory conditions such as ARDS involve more complex pathophysiological processes, including inflammation-induced alveolar-capillary barrier disruption and surfactant inactivation. Despite early biophysical enthusiasm, several large-scale clinical trials of systemic exogenous surfactant therapy in adult ARDS populations failed to demonstrate consistent mortality benefits, leading to a temporary decline in clinical interest. As highlighted by [6], systemic exogenous surfactant administration has failed to demonstrate a significant mortality benefit in generalized adult ARDS cohorts. This underscores the reality that extrapolating findings from neonatal models or controlled animal studies to the highly complex, protein-rich inflammatory environment of the adult post-thoracotomy lung is inherently limited. However, recent studies suggest that factors such as timing of administration, mode of delivery, and patient selection are critical variables influencing outcomes [52–53]. Beyond ARDS, surfactant dysfunction has also been implicated in perioperative pulmonary complications in patients undergoing complex thoracic procedures, including cardiac surgery and lung transplantation. In these settings, alveolar de-recruitment, ischemia-reperfusion injury, and inflammatory responses can impair surfactant synthesis and activity [54–55]. Early pilot studies and case series have demonstrated that prophylactic or rescue administration of exogenous surfactant may improve intraoperative oxygenation and reduce the incidence postoperative pulmonary complications [56–59]. However, these findings are based on limited sample sizes and non-randomized designs, and thus require validation through large-scale, controlled clinical trials.

## Perioperative applications of surfactants in thoracic surgery

### The proactive window for alveolar stabilization

Thoracic surgical procedures often require partial or complete lung deflation, use of OLV, and prolonged manipulation of lung tissue. These interventions mechanically displeace endogenous surfactants, directly predisposeing the alveoli to collapse, and impaired gas exchange [60]. These adverse effects are particularly amplified during major procedures such as lung, mediastinal lession, thoracic esophageal carcinoma resection, where intraoperative pulmonary stress can induce ventilation-induced lung injury (VILI), characterized by ischemia–reperfusion injury, and inflammatory activation [62]. Given their biophysical capacity to reduce surface tension and stabilize alveoli, surfactants present an attractive adjunct for perioperative use. Unlike the reactive treatment of established medical ARDS, the operating room offers a unique “proactive” window. Administerign surfactant prior to or immediately during lung re-expansion may has the potential to maintain alveolar patency, prevent atelectasis, and improve lung compliance, thereby facilitating effective re-expansion of the lung after deflation and optimizing postoperative respiratory function [61]. In animal models, intraoperative instillation of surfactant has been shown to reduce pulmonary edema, improve dynamic compliance, and enhance oxygenation following prolonged OLV or ischemia–reperfusion injury [62–55]. Similarly, in clinical pilot studies involving thoracic aortic aneurysm surgery, intraoperative or early postoperative surfactant administration was associated with better gas exchange and reduced incidence of ventilator-associated complications [4].

### Intraoperative applications of surfactants

Delivery routes for intraoperative surfactant administration include direct tracheobronchial instillation, aerosolized nebulization, or even topical application to exposed lung surfaces during surgery [63–64]. Among these, bronchoscopic instillation into dependent lung segments has shown promising, particularly in open-chest procedures or transplant settings [65–66]. Furthermore, timing appears to be a critical determinant of efficacy: prophylactic administration before lung re-expansion or early rescue after ischemic stress may provide the greatest benefit [67–68]. In addition to direct mechanical effects, surfactants are being explored as multifunctional vehicles for intraoperative drug delivery. They may serve as carriers for anti-inflammatory agents or antibiotics, thereby offering dual-function benefits in modulating immune responses or preventing infection in high-risk surgical fields [69–70]. Surfactant-based lavage techniques are also being investigated for intraoperative pulmonary decontamination, particularly in cases of aspiration or infected pleural spaces [71–72]. Novel diagnostic applications of surfactant-based materials have emerged. For example, Yang et al. developed a 15% Pluronic F-127 surfactant-based bubble solution for air leak detection during video-assisted thoracoscopic surgery (VATS), demonstrating good visibility and procedural feasibility [73]. Although not therapeutic, this underscores the expanding role of surfactant-like compounds in thoracic operative settings.

When evaluating the intraoperative application of exogenous surfactants, it is critical to distinguish between translational hypotheses and actual clinical evidence. Challenges include standardization of dosage, formulation, and delivery protocols, as well as defining optimal clinical indications. In particular, it remains to be clarified which patients derive the greatest benefit from intraoperative surfactant intervention. Therefore, the current benefit of intraoperative surfactant therapy should be realistically framed as a localized, symptom-resolving adjunct for specific mechanical complications, rather than a generalized cure. Multicenter randomized controlled trials still need to establish evidence-based guidelines and further integrate surfactant therapy into thoracic surgical practice.

### Postoperative consideration: translational concepts and clinical limitations

In the postoperative period following thoracic surgery, patients are at heightened risk for pulmonary complications, including atelectasis, impaired lung compliance, and ARDS. These conditions are often precipitated by alveolar surfactant depletion or inactivation due to factors including mechanical ventilation, ischemia–reperfusion injury, systemic inflammatory responses, or the mechanical disruption of surfactant films by surgical irrigation and suctioning [74–75]. In selected experimental and early clinical studies, postoperative administration of surfactant has been associated with improvements in oxygenation and ventilator parameters, although direct evidence of reduced lung injury on imaging or histopathology remains limited [76]. Administering surfactant early in the postoperative course, particularly in patients exhibiting early signs of hypoxemia or radiographic infiltrates, may mitigate disease progression. Some studies have proposed using serial PaO_2_/FiO_2_ ratios and dynamic lung measurements metrics to guide individualized surfactant therapy in the surgical intensive care settings [77–78]. Notably, the benefits of surfactant may extend beyond biophysical effects; certain surfactant proteins—particularly SP-A and SP-D—exert direct antimicrobial activity and enhance alveolar macrophage-mediated clearance of pathogens [79–80]. Beyond mechanical benefits, surfactants may play a role in modulating immune responses and preventing postoperative infection. There is increasing interest in postoperative use of surfactant-enhanced antibiotics or anti-inflammatory agents to target biofilm-forming organisms in pleural infections or contaminated surgical drains [81–83].

However, challenges remain. In adults, especially after major thoracic surgery, the effectiveness of exogenous surfactant has shown mixed results. Factors such as heterogeneous intrapulmonary distribution, rapid inactivation in inflamed alveoli, and interpatient variability in response may limit therapeutic benefit [84–86]. Additionally, some studies report transient hypoxemia or ventilation–perfusion mismatch following instillation, likely due to surfactant bolus redistribution [87–88]. Moreover, unlike neonates with primary deficiency, postoperative ARDS in adults often arises in the context of pre-existing lung injury and systemic inflammation, which may further compromise surfactant function and bioavailability [36–37]. Patients at particular risk—such as those with chronic pulmonary disease, prolonged intraoperative one-lung ventilation, or early postoperative radiographic infiltrates—may represent optimal candidates for surfactant-based interventions. Ongoing research into improved formulations, including aerosolized or liposomal surfactants, and more precise dosing strategies, is essential to overcoming current barriers and unlocking the full therapeutic potential of surfactant therapy in the postoperative setting.

## Drug delivery and surfactant-based systems

Beyond their classical biophysical functions in reducing alveolar surface tension, pulmonary surfactants have garnered growing interest as versatile platforms for targeted drug delivery, particularly within the respiratory system [89]. For instance, in a neonatal piglet model of hydrochloric acid-induced ARDS, Huang et al. showed that co-administration of lidocaine with surfactant during lavage improved lung mechanics and gas exchange more effectively than surfactant alone [90]. Due to their amphiphilic character, compatibility with epithelial surfaces, and ability to form lipid-rich structures, surfactants are particularly suitable for delivering therapeutic agents to the alveoli and pleural spaces [91–92]. These systems offer a distinct advantage for pulmonary delivery of hydrophobic drugs, which often suffer from poor solubility and limited tissue penetration. Incorporating such agents into surfactant-based liposomes or micelles can enhance drug stability, extend residence time in the airways, and enable controlled release at the target site [93]. This is largely attributed to the ability of surfactant vesicles to shield encapsulated drugs from enzymatic degradation and mucociliary clearance mechanisms. Liposomal encapsulation—particularly using surfactant-derived phospholipids such as DPPC or phosphatidylglycerol—creates a biocompatible and biodegradable matrix that prolongs drug half-life in the alveolar milieu [94]. Moreover, these nanoscale carriers exhibit preferential deposition in the peripheral airways due to their aerodynamic properties, improving local bioavailability while minimizing systemic exposure [95]. Notably, when administered via aerosol, pulmonary surfactants can act as functional carriers that optimize drug deposition by reducing surface tension and improving particle dispersion throughout the alveolar tree [96]. This “aerosol carrier” behavior facilitates deeper pulmonary penetration and more uniform drug distribution, particularly into distal and collapsed regions of the lung where conventional aerosols often fail to reach. Surfactants also improve the aerodynamic behavior of inhaled particles by modulating viscosity and particle aggregation, thus reducing impaction losses in the upper airways and enhancing alveolar bioavailability [94]. Such findings underscore the growing relevance of surfactant-based aerosol systems in both acute and chronic respiratory therapies.

In thoracic surgery and pulmonology, several potential applications have emerged: (1) Lung cancer therapy: Lipid-based surfactants can be used to encapsulate chemotherapeutic agents such as paclitaxel or cisplatin. These particles can be delivered via nebulization or intratracheal instillation, allowing high local drug concentrations with minimal systemic toxicity [97–98]. Surfactant films also improve the dispersion and deposition of inhaled particles deep into peripheral lung segments [99]. (2) Anti-inflammatory applications: Corticosteroids or other immunomodulatory agents can be incorporated into surfactant mixtures to treat postoperative pneumonitis or acute lung injury [100–101]. Surfactant-based carriers improve epithelial absorption and reduce enzymatic degradation, prolonging drug efficacy [102]. (3) Antimicrobial delivery: Surfactants can facilitate the delivery of antibiotics or antiseptics into infected pleural or parenchymal tissue, such as in empyema or postoperative pneumonia [103–104]. Some surfactant proteins, like SP-A and SP-D, possess intrinsic antimicrobial activity, enhancing alveolar macrophage-mediated pathogen clearance [79–105]. (4) Pleural and peritoneal lavage systems: Surfactant-containing lavage fluids have been proposed to improve distribution in pleural or peritoneal spaces, particularly in loculated effusions or contaminated surgical fields [106–107]. These systems may also act as anti-adhesive barriers by coating serosal surfaces and minimizing postoperative adhesion formation [108].

Emerging technologies are developing next-generation surfactant-based delivery systems, including nanoparticle-bound surfactants, enzyme-sensitive formulations, and bioresponsive conjugates capable of releasing cargo in response to pH changes or inflammatory markers [109–112]. In thoracic surgery, these strategies may offer perioperative advantages such as modulating wound healing, limiting fibrosis, and targeting residual tumor cells at resection margins. Despite these promising developments, key challenges remain in formulation stability, drug loading efficiency, manufacturing scalability, and regulatory approval pathways. Despite compelling preclinical evidence and the growing sophistication of surfactant-assisted drug delivery platforms, clinical translation remains limited. Several factors contribute to this gap. First, the complexity of surfactant-drug interactions—particularly concerning stability, aggregation, and bioavailability—poses formulation challenges that are difficult to standardize across patient populations and manufacturing batches. Second, differences in respiratory physiology between animal models and human lungs, especially in disease states such as ARDS or postoperative inflammation, often hinder the predictive value of preclinical outcomes. Finally, logistical and economic barriers—including the need for specialized delivery devices and the cost of high-purity or synthetic surfactants—have limited widespread implementation. Bridging this gap requires multidisciplinary efforts to develop scalable, reproducible formulations with well-defined pharmacokinetic profiles. Clinical trials must be carefully designed to identify patient subgroups most likely to benefit or patients undergoing major thoracic surgery. With rigorous translational research and regulatory guidance, surfactant-based drug delivery systems hold the potential to move from experimental innovation to clinical reality.

## Future perspectives: biocompatible, bio-derived surfactants

Conventional surfactants used in medical practice - particularly those derived from animal sources or petroleum-based chemicals - have demonstrated significant efficacy, yet are not without limitations. Issues such as batch to batch variability, immunogenicity, environmental impact, and dose dependent cytotoxicity have prompted increasing interest in next-generation surfactants that are safer, more consistent, and sustainably produced [113–114]. In response, recent advancements in biocompatible and bio-derived surfactants offer promising alternatives. These include surfactants synthesized from amino acids, sugar esters, fatty acids, and microbial fermentation products [115]. Their amphiphilic structures mimic endogenous phospholipids while offering enhanced biodegradability, lower toxicity, and chemical flexibility for functionalization—enabling integration with therapeutic agents or targeting ligands [116–118]. Notably, several bioengineered surfactant formulations have entered advanced preclinical or clinical evaluation. CHF5633, a fully synthetic surfactant composed of DPPC, palmitoyl-oleoyl phosphatidylglycerol, and two SP-B and SP-C mimetic peptides, has demonstrated improved biophysical properties and anti-inflammatory activity in ARDS models [119]. Similarly, Venticute, a recombinant SP-C–containing formulation, has shown enhanced resistance to inactivation by serum proteins and was evaluated in phase II trials for sepsis-associated ARDS [120]. This is critical in thoracic surgery, where the alveolar space is often compromised by the protein-rich inflammatory exudate and hemorrhage characteristic of major lung resections and OLV. Beyond peptide-based systems, biosurfactants such as rhamnolipids—produced by Pseudomonas aeruginosa—and sophorolipids, derived from yeast fermentation, offer promising lung compatibility and bioactivity, including antibacterial and immunomodulatory effects [121]. Recent studies have also explored lipopeptides such as surfactin, a cyclic biosurfactant from Bacillus subtilis, for their ability to form stable monolayers and enhance mucus dispersion in pulmonary applications [122].

In thoracic surgery, bio-derived surfactants may offer several distinct advantages over conventional formulations: (1) Improved safety profile for intraoperative and postoperative administration, particularly in inflamed, injured or immunocompromised lungs. (2) Prolonged alveolar retention, attributed to improved structural stability under mechanical stress. (3) Customizable molecular structures, allowing integration with antibiotics, anti-adhesive agents, or tumor-targeting ligands. (4) Reduced immunogenicity, a particularly relevant feature in lung transplant recipients or patients receiving ECMO support. A particularly exciting innovation lies in the use of biosurfactants derived from microbial sources, such as rhamnolipids, sophorolipids, and lipopeptides. These naturally occurring compounds have shown excellent surface activity and compatibility with lung epithelium, alongside intrinsic antimicrobial, anti-inflammatory, and immunomodulatory properties [123–127]. Such features are highly desirable for use in perioperative lung protection or lavage therapies during thoracic procedures.

Another area of active research is the development of stimuli-responsive surfactants—engineered molecules designed to alter their physicochemical behavior in response to local microenvironmental cues such as pH, protease activity, or inflammatory markers [128]. These advanced delivery systems may enable spatially selective drug release in infected, ischemic, or tumor-infiltrated lung regions, thereby minimizing systemic toxicity while maximizing therapeutic efficacy. Despite these promising developments, several translational challenges must be addressed: (1) Scalable and reproducible manufacturing methods for structurally complex surfactant systems. (2) Regulatory pathways for approval of novel bioactive surfactants and surfactant–drug conjugates. (3) Integration with existing thoracic surgical tools and protocols, including nebulizers, bronchoscopic delivery platforms, and ECMO circuits. (4) Comprehensive safety, immunogenicity, and pharmacokinetic profiling in preclinical and early-phase clinical models, particularly in the context of human lung transplantation and injury. Progress in this field will require close collaboration among materials scientists, pulmonologists, thoracic surgeons, and pharmaceutical developers. Ultimately, well-designed clinical trials will be essential to establish optimal formulations, delivery routes, and therapeutic indications for these emerging surfactant-based platforms in thoracic medicine.

## Conclusion

In conclusion, while the biochemical engineering of exogenous surfactant formulations continues to advance, their routine application in adult thoracic surgery remains investigational. The biophysical capacity of surfactants to reduce alveolar surface tension provides a compelling theoretical framework for stabilizing the air-liquid interface and mitigating perioperative atelectrauma. However, it must be clearly stated that solid, real-world clinical evidence demonstrating definitive perioperative benefits is currently lacking. With sustained interdisciplinary innovation and well-designed clinical trials, surfactant-based interventions may evolve into valuable adjuncts for improving perioperative respiratory outcomes and long-term recovery in thoracic surgery.

## Data Availability

No datasets were generated or analysed during the current study.
